# Navigating the fear: assessing nursing students’ concerns and preventive practices in response to Monkeypox in Egypt

**DOI:** 10.1186/s12912-024-02589-2

**Published:** 2025-01-07

**Authors:** Shaimaa Mohamed Amin, Doaa El Demerdash, Mona Metwally El-Sayed, Tasneem Ragab Ahmed Salama, Mohamed Gamal Elsehrawy, Mohamed Hussein Ramadan Atta

**Affiliations:** 1 Community Health Nursing, Faculty of Nursing, Damanhour University, Damanhour, Egypt; 2 Nursing Education Department, Faculty of Nursing, Damanhour University, Damanhour, Egypt; 3 Faculty of Applied Health Sciences Technology, Galala University, Galala, Egypt; 4 Psychiatric and Mental Health Nursing, Faculty of Nursing, Alexandria University, Alexandria, Egypt; 5 Community Health Nursing, Faculty of Nursing, Beni-Suef University, Beni-Suef, Egypt; 6 Nursing Administration Department, Faculty of Nursing, Port Said University, Port Said, Egypt; 7 Psychiatric and Mental Health Nursing, Faculty of Nursing, Alexandria University, Alexandria, Egypt

**Keywords:** Monkeypox, Nursing students, Fear, Concerns, Preventive practices, Health education, Egypt, Cross-sectional study

## Abstract

**Background:**

Monkeypox has emerged as a global health concern, necessitating preventive measures and awareness among healthcare students. Understanding nursing students’ fears, concerns, and preventive practices regarding Monkeypox can help improve preparedness and response strategies.

**Aim:**

This study aimed to assess the levels of fear, concerns, and preventive practices related to monkeypox among nursing students at Beni Suef University, Egypt, during the 2024–2025 academic year.

**Methods:**

A cross-sectional descriptive design was employed, involving 505 nursing students systematically randomized recruited. Data collection utilized three validated tools: the Monkeypox Fear Scale, Monkeypox Concern Scale, and Monkeypox Preventive Practices Scale. Reliability was confirmed with Cronbach’s alpha values ranging from 0.87 to 0.94. Data analysis included descriptive statistics, Spearman correlation, and multiple linear regression using SPSS Version 23.

**Results:**

Most (56.2%) students rarely worried about Monkeypox infection, though 49.2% reported anxiety influenced by media coverage. High preventive practices were noted, with 60.6% frequently practicing hygiene measures. A strong positive correlation between Monkeypox concern and fear (*r* = 0.646, *p* < 0.001) and a moderate positive correlation between Monkeypox concern and preventive practices (*r* = 0.229, *p* = 0.001). Fear was also significantly correlated with preventive practices (*r* = 0.432, *p* < 0.001). Multiple linear regression analysis (R² = 0.216, F = 22.633, *p* < 0.001) revealed that fear was a strong positive predictor of preventive practices, while Monkeypox concern had a positive but non-significant effect (B = 0.138, *p* = 0.156). Age was not a significant predictor (B = 0.251, *p* = 0.637), whereas family income showed a significant negative association (B = -1.885, *p* = 0.010).

**Conclusion:**

The study revealed moderate fear and concerns among nursing students, with generally high adherence to preventive practices.

**Implication:**

Findings suggest the need for targeted health education programs to address monkeypox-related concerns and enhance preventive measures, thus improving nursing students’ readiness to respond to emerging health threats.

## Introduction

Monkeypox (Mpox), a zoonotic viral disease caused by the Monkeypox virus (MPXV), has become a significant public health concern worldwide. The global monitoring reported 102,977 confirmed cases and 219 deaths across 121 countries, with most cases attributed to MPXV clades I and II. In Africa, where the disease remains most prevalent, over 20,000 cases were recorded in 13 African Union member states, with a case fatality rate of 2.9%. This highlights the ongoing need for enhanced surveillance, preventive strategies, and targeted public health interventions to control Mpox’s spread and mitigate its impact [1–3].

As future healthcare professionals, nursing students are not just participants but pivotal in responding to global health emergencies like the Mpox virus outbreak [4]. This virus, documented in numerous countries, has raised significant public health concerns [5]. The Africa Centres for Disease Control and Prevention (ACDC) declared the Monkeypox Virus Clade I (MPXV), also called Mpox, a Public Health Emergency of Continental Security [6]. The following day, the WHO convened and classified the MPXV Clade I outbreak as a Public Health Emergency of International Concern [5].

During outbreaks, nursing students may experience heightened fear and anxiety, which can significantly hinder their ability to implement proper preventive measures [7]. Recognizing and addressing their levels of worry and concern is crucial, as these emotional responses can significantly influence their decision-making, adherence to infection control protocols, and overall preparedness to handle emerging health crises. Therefore, providing students with the necessary education, resources, and psychological support is essential to mitigate these concerns and enhance their readiness to face future emergencies effectively, instilling the confidence to handle such situations.

Monkeypox, an orthopoxvirus, is responsible for causing Mpox, a zoonotic disease transmitted from animals to humans. The virus is typically found in infected animals near tropical rainforests, including squirrels, Gambian pouched rats, dormice, and monkey species. It is crucial to note that human-to-human transmission is also possible through various means, including direct contact with bodily fluids, skin lesions, internal mucosal surfaces such as the mouth or throat, respiratory droplets, and contaminated items [8, 9].

Monkeypox shares similarities with smallpox but is generally less severe. Although smallpox was eradicated in 1980, it persists in central and western Africa. Since May 2022, cases have also been reported in countries outside of Africa with no history of Mpox transmission [10]. The Mpox has two distinct clades: Clade I, previously called the Congo Basin (central African) Clade, and Clade II, formerly the West African Clade [11].

As of July 31, 2024, global Mpox monitoring recorded 102,977 confirmed cases and 219 deaths across 121 countries, primarily caused by MPXV clade I and II. The European CDC (ECDC) reported over 20,000 cases in 13 African Union member states, with a case fatality rate of 2.9%. The Democratic Republic of Congo (DRC) accounted for the highest number of cases (19,667) and deaths (575), with most cases (66%) and deaths (82%) occurring in individuals under 15. Notably, Mpox clade I cases have been predominantly reported in Africa, except for isolated cases in Sweden and Thailand [1–3].

The WHO Director-General has declared the Mpox outbreak a public health emergency of international concern [5]. The social reaction to the spread of Mpox remains uncertain. One of the critical psychological responses to such outbreaks is fear, a powerful emotion that significantly impacts both physiology and behavior. Fear is typically triggered when an individual perceives a looming threat to their survival [12]. Research has explored fear’s biological, evolutionary, and social underpinnings, noting both individual and collective responses. Neurobiologically, fear is processed through specific neural pathways, particularly involving the amygdala, hypothalamus, and brainstem circuits. These systems monitor threats and trigger defense mechanisms aimed at self-preservation [13–15].

From a psychological perspective, fear is typically seen as an adaptive, short-term reaction that dissipates once the threat is no longer present [16]. For healthcare providers, it is crucial to examine emotional responses to emerging threats like Monkey pox, as these reactions can impact their psychological well-being and influence their professional duties during outbreaks. The current situation has raised an international alert for governments and national health systems due to the increased demand for medical care. Several studies have begun exploring the attitudes, knowledge, and preventive practices related to Mpox. Initial findings indicate that awareness of Mpox was relatively low among healthcare professionals and the public, at least until the end of May [17, 18]. In addition, Baiocco [19] found that increased fear of Mpox was linked to higher levels of epistemic credulity, close-mindedness, anxiety, and difficulties in expressing and processing emotions. On the other hand, lower fear levels were associated with the belief that the media had exaggerated the risks of Mpox outbreaks.

Preventative measures against Mpox, a viral zoonotic disease, are crucial to control its spread, particularly during outbreaks [20]. These measures include vaccination, proper use of personal protective equipment (PPE), isolation of infected individuals, and adherence to hygiene practices such as handwashing and disinfecting contaminated surfaces. The importance of these measures cannot be overstated for healthcare providers, as they are on the frontlines of managing outbreaks and are at higher risk of exposure [21, 22]Effective prevention strategies protect healthcare workers and minimize the potential for widespread transmission within healthcare settings. Lulli et al. (2022) concluded that the Key recommendations for preventing and managing Mpox in workplace environments include vaccinating exposed workers, promptly identifying and isolating infected individuals, and maintaining proper hygiene practices. In non-endemic countries, healthcare workers should receive education and specialized training to help them recognize the disease and prevent its spread.

However, fear associated with Mpox outbreaks can significantly influence the adoption of these preventative measures. Higher levels of fear can lead to more vigilant behaviors, such as increased use of PPE and stricter adherence to protocols [23]. On the other hand, fear might also cause anxiety or reluctance among healthcare workers, potentially impacting their ability to respond effectively. The MPX outbreak and the implementation of containment measures, such as social isolation, quarantine, and self-isolation, have been shown to impact mental health significantly. Isolation and lack of social interaction are known to exacerbate psychiatric disorders, including depression and schizophrenia, as these conditions thrive in environments devoid of human contact. Prolonged isolation increases the likelihood of developing or worsening depression and anxiety [23].

Research from previous pandemics, particularly during quarantine, reveals that healthcare professionals who self-isolated were at heightened risk of substance use disorders, post-traumatic stress disorder (PTSD), and depression [24]. Therefore, it is essential for healthcare providers treating MPX patients to receive consistent mental health training to help prevent the onset of these disorders. Moreover, Mpox and smallpox infections in humans present serious risks, including miscarriage, severe infections, and maternal or fetal mortality. The known vertical transmission of these viruses increases psychological stress in pregnant or breastfeeding women, which may contribute to higher abortion rates [3]. In addition, Alder et al. 2022 identified seven new MPX cases, three of which involved patients reporting low mood and uncertainty about their condition. This suggests that the 2022 MPX outbreak has had a notable effect on the mental health of those infected [25]. Given these findings, it is critical to assess and address the fear and concern surrounding Mpox to mitigate its psychological impact.

Current research on Mpox has primarily focused on healthcare professionals, the general public, and workplace environments, addressing their levels of awareness, fear, and adherence to preventive measures [17, 18, 20]. However, a notable gap exists in understanding how nursing students, as future healthcare providers, perceive the risks associated with Mpox, including their level of fear and concern. While nursing students are critical to the healthcare workforce, there is a lack of research examining their preparedness to adopt preventive measures against Mpox and the influence of their educational background on these behaviors. Additionally, the psychological stress associated with Mpox, such as fear of infection, isolation, and its impact on mental health, has not been sufficiently studied in this population, particularly concerning its effects on their academic performance and overall well-being. Addressing this gap through research would provide valuable insights into nursing students’ experiences and help guide targeted training and mental health interventions to equip them for future outbreaks better. This study aimed to assess nursing students’ concerns and preventive practices in response to the emerging threat of Mpox. It seeks to understand how nursing students perceive the risk, their levels of fear, and the measures they are taking to prevent infection to identify gaps and inform the development of targeted educational interventions.

## Objectives of the study

1. To assess the level of concern among nursing students regarding Mpox.
2. To explore nursing students’ fears about Mpox and its impact on their academic and clinical training.
3. To evaluate nursing students’ preventive practices in response to Mpox.
4. To identify the factors associated with nursing students’ concerns and preventive behaviors related to Mpox.

## Methods

### Study design

The study utilized a cross-sectional descriptive research design, adhering to the Strengthening the Reporting of Observational Studies in Epidemiology (STROBE) guidelines.

### Setting

The study was conducted at the Faculty of Nursing at Beni Suef University in Beni Suef Governorate, Egypt. The Egyptian Ministry of Higher Education oversees the faculty and complies with national nursing education standards. The faculty consists of nine specialized departments, each focusing on different aspects of nursing. The undergraduate and graduate programs operate on a credit-hour system, offering a well-structured method for monitoring academic progress and rigorously evaluating educational outcomes.

### Sample size and study participants

In this study, the inclusion criteria were nursing students currently enrolled in the Faculty of Nursing at Beni Suef University for the 2024–2025 academic year who were willing to participate. Exclusion criteria included individuals not enrolled in a nursing program, those with diagnosed mental health conditions that could affect their perception of fear and concerns, and those unwilling to participate in the study.

**Fig. 1:**
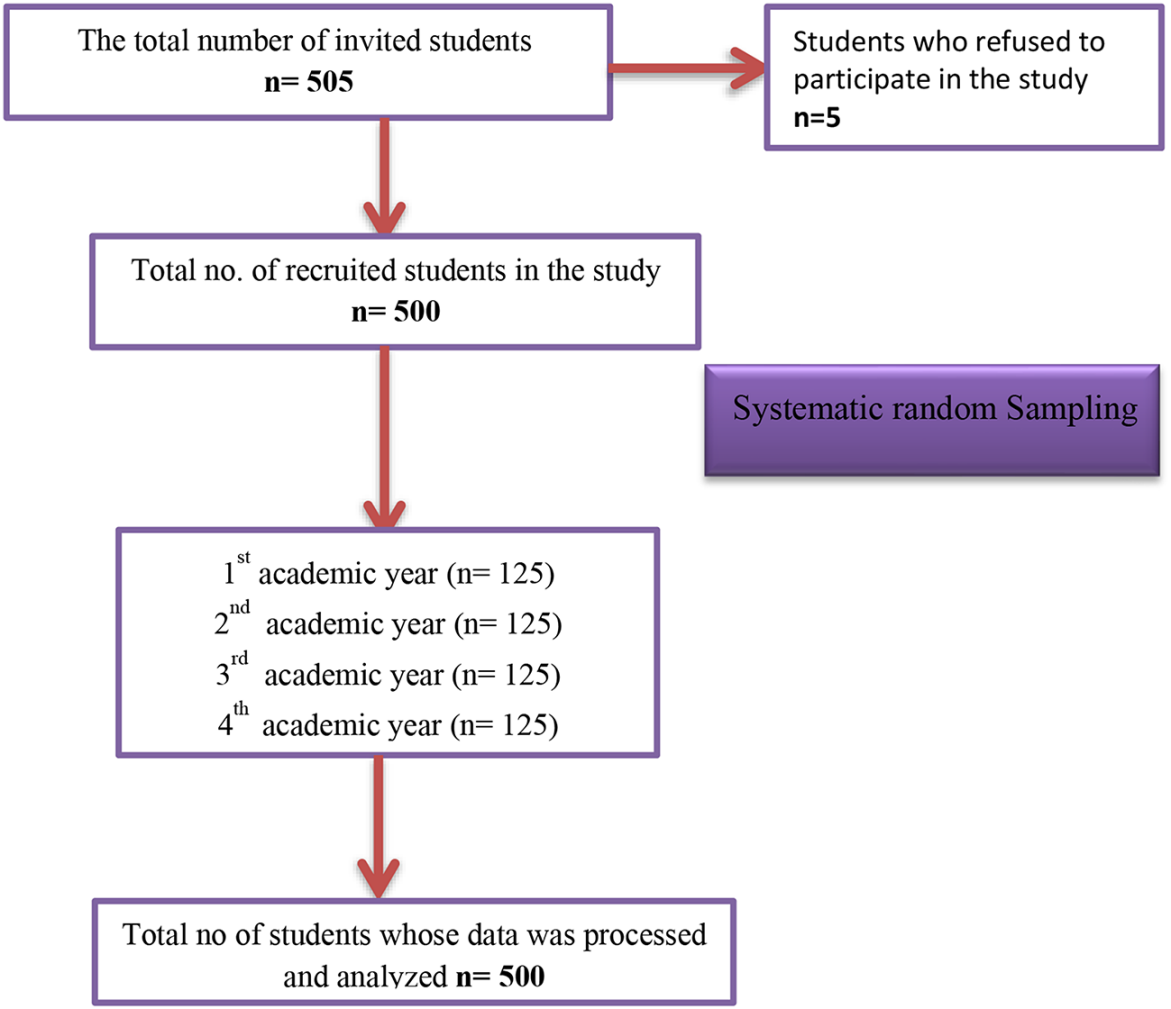
Flow Chart of Participants’ Recruitment

We employed the G* Power version 3.1.9.7 software program developed by Erdfelder [26] to calculate the sample size. The specified parameters for the calculation included an estimated effect size of 0.15, α error probability of 0.05, and Power (1-β error probability) of 0.90. The program recommended a minimum total sample size of 459. However, the researchers decided to recruit 505 students in case of the possibility of withdrawal, dropout rates, or the incompleteness of the study tools. However, five students refused to participate, so the final sample size was 500, as shown in Fig. (1).

### Measurements of interest

#### Demographic form

The form collects socio-demographic information, including age, sex, academic year, place of residence, family type, income, marital status, and parents’ educational level.

### Monkeypox Fear Scale (MFS)

Caycho-Rodríguez et al. (2022) [27] developed the Monkeypox Fear to assess fear levels related to Mpox. It includes seven items with five Likert-type response options ranging from 1 (strongly disagree) to 5 (strongly agree), where higher scores indicate greater fear. The scale exhibited strong content validity, with Aiken’s V values above 0.70 for clarity, coherence, and relevance. Confirmatory Factor Analysis (CFA) supported a two-factor model with satisfactory fit indices (CFI = 0.99, TLI = 0.99, RMSEA = 0.074). The scale also demonstrated high reliability, with omega coefficients of 0.91. The scale was translated into Arabic in this study, and its reliability was confirmed with a Cronbach’s alpha of 0.87. Exploratory factor analysis showed improved factor loadings after rotation, explaining 72.354% of the total variance.

### Monkeypox concern scale

The Monkeypox Concern Scale was adapted initially and validated by Mamani-Benito, Carranza Esteban [28], is designed to assess individuals’ levels of concern regarding Mpox. Comprising six items, this scale uses a four-point Likert scale ranging from “never” (1) to “almost all the time” (4). The total score ranges from 6 to 24, with higher scores indicating more significant concern or anxiety about Mpox. The scale demonstrates robust validity and reliability. Construct validity was confirmed through confirmatory factor analysis, which revealed a good model fit with indices such as CFI = 0.96, TLI = 0.94, RMSEA = 0.08 (95% CI [0.067–0.097]), and SRMR = 0.03. The internal consistency of the scale, measured by the omega coefficient, was found to be 0.89, reflecting a high level of reliability. The current study reported the scale’s reliability coefficient was reported as 0.92, further indicating excellent internal consistency. An exploratory factor analysis (EFA) was also conducted to validate the instrument following its translation from English to Arabic. Factor loadings ranged from 0.530 to 0.870 before rotation and improved to 0.650 to 0.960 after varimax rotation, exceeding the 0.40 threshold. These factors collectively explained 72.4% of the total variance, affirming the scale’s strong construct validity.

### Monkeypox preventive practices scale

In the study by Díaz-Ortega [29], the Preventive Practices Scale was designed to evaluate behaviors related to Mpox prevention among citizens in Peru. This scale, consisting of 9 items, was divided into two dimensions: preventive practices concerning individual hygiene (4 items) and environmental preventive practices (5 items). Participants rated each item on a five-point Likert scale, from never (1) to always (5), with higher scores indicating greater adherence to preventive measures. The scale demonstrated internal solid consistency, supported by Cronbach’s alpha and McDonald’s omega values. Specifically, the “Individual Hygiene” subscale had a Cronbach’s alpha of 0.8891 and McDonald’s omega of 0.8977, while the “Prevention in the Environment” subscale showed a Cronbach’s alpha of 0.8666 and McDonald’s omega of 0.8830. The overall “Practices” dimension had high reliability, with a Cronbach’s alpha of 0.9154 and McDonald’s omega of 0.9315, reflecting the scale’s effectiveness in measuring preventive practices. Furthermore, the preventive practices scale exhibited strong content and construct validity, as demonstrated by exploratory factor analysis. Correlation analyses indicated high associations among items within the dimensions, with the scale of the practice showing correlations up to 0.78. This study reported the scale’s reliability coefficient as 0.94, indicating excellent internal consistency. EFA revealed factor loadings ranging from 0.600 to 0.850 before and 0.700 to 0.970 after varimax rotation, exceeding the 0.40 threshold. These factors accounted for 75.2% of the total variance, affirming the scale’s robust construct validity.

### Study procedures

#### Tool preparation and pilot study

The research instruments, including the Monkey Pox Fear Scale, Monkey Pox Concern Instrument, and Monkey Pox Preventive Practices Scale, were carefully translated into Arabic by bilingual experts fluent in English and Arabic. Great emphasis was placed on ensuring accuracy and cultural relevance in the translations. To validate these translations, they were back-translated into English to verify linguistic equivalence and resolve any inconsistencies. Following translation and back-translation, each instrument underwent face validity assessments. Expert panels thoroughly reviewed the translated tools to confirm they effectively captured the intended constructs within the Arabic context. Additionally, feedback was gathered from potential participants to evaluate the clarity, relevance, and cultural appropriateness of the translated items. Reliability was assessed using statistical methods, including Cronbach’s alpha, to confirm internal consistency. A pilot study with 50 nursing students was conducted to test the translated instruments’ clarity, relevance, and reliability. These participants were subsequently excluded from the main study. The pilot study results showed that no modifications were needed for the tools.

### Data collection

Data collection took place between August and September 2024, beginning with a thorough orientation session led by the researcher. During this session, the study’s objectives were clearly explained, emphasizing the voluntary nature of participation. Participants were encouraged to ask questions, and any concerns were promptly addressed. Confidentiality protocols were also reinforced to ensure trust in the research process. The questionnaires were distributed to students in various locations, including lecture halls and libraries, from Sunday to Thursday between 10 a.m. and 2 p.m. Both verbal and written informed consent was required for participation. On average, participants spent 10–15 min completing the questionnaire.

### Ethical considerations

The study was approved by the Research Ethics Committee of the Faculty of Nursing at Beni Suef University, Egypt, under reference number (FMBSUREC/01102024/Salama). All procedures adhered strictly to the ethical standards outlined in the Helsinki Declaration. Before participation, each individual was given comprehensive information regarding the study’s objectives and procedures. Written informed consent was obtained from every participant. The research maintained a solid commitment to ethical principles, including voluntary participation, anonymity, and confidentiality. Participants were explicitly informed of their right to withdraw from the study at any time without any repercussions.

### Data analysis

SPSS software was used to conduct the statistical analysis (Statistical Package for Social Science, Version 23). The demographic characteristics were described using number and percentage statistics depending on the level of measurement. Mean and standard deviation were employed as descriptive statistics to define the scores of Mpox concerns, fear, and preventive practices used by nursing students. Spearman correlation test was used to determine and measure the correlation between different study variables. To determine factors predicting and affecting good preventive practices against Mpox using multiple linear regression analysis. The demographic variables (age, sex, parents’ educational level, etc.) were included in the model, using alpha 0.05, along with the predictors.

## Results

Table 1 reveals that those under 20 reported lower levels of concern and fear regarding Mpox compared to older age groups, with those aged 22–24 exhibiting slightly higher fear levels (25.38 ± 5.53). Interestingly, preventive practices tended to increase with age, suggesting that older participants may be more proactive in engaging in health-protective behaviors. Individuals reporting insufficient income expressed higher levels of concern (13.57 ± 4.78) and fear (25.47 ± 6.12) about Mpox compared to those whose income was sufficient for basic needs or savings. Furthermore, marital status influenced concern levels, with single individuals generally reporting lower concerns than those who were divorced or widowed. However, higher parental education correlated with lower levels of fear and higher preventive practices (*p* < 0.001), indicating that education may enhance awareness and understanding of health issues. No significant relation was found between gender, region of residence, and type of family with study variables.

**Table 1:** Demographic Characteristics of the participants and relation with Monkeypox Concern, fear, and preventive practices (*n* = 500)

Variable	*N*	%	Mpox Concern	MFS	Preventive practices
**Age**					
< 20	190	38.0	11.47 ± 6.01	20.98 ± 8.59	22.87 ± 11.08
20 < 22	208	41.6	11.58 ± 5.46	24.41 ± 6.14	28.36 ± 8.81
22–24	86	17.2	11.63 ± 3.93	25.38 ± 5.53	26.47 ± 8.13
≥ 24	16	3.2	12.38 ± 4.65	24.06 ± 5.82	26.88 ± 8.97
**F (p)**			**0.143 (0.934)**	11.016 (< 0.001*)	10.894 (< 0.001*)
**Gender**					
Male	268	53.6	11.49 ± 5.68	23.41 ± 7.23	25.89 ± 9.71
Female	232	46.4	11.67 ± 5.12	23.09 ± 7.37	25.91 ± 10.19
**t (p)**			**0.388 (0.698)**	**0.482 (0.630)**	**0.020 (0.984)**
**Region of Residence**					
Rural	296	59.2	11.41 ± 5.47	23.20 ± 7.79	25.85 ± 9.84
Urban	204	40.8	11.80 ± 5.35	23.36 ± 6.51	25.97 ± 10.07
**t (p)**			**0.794 (0.428)**	**0.260 (0.795)**	**0.132 (0.895)**
**Family Monthly Income**					
Insufficient	76	15.2	13.57 ± 4.78	25.47 ± 6.12	26.16 ± 9.87
Sufficient basic needs only	306	61.2	11.52 ± 5.48	24.15 ± 6.47	28.31 ± 8.43
Sufficient and save	118	23.6	10.42 ± 5.35	19.54 ± 8.62	19.48 ± 10.75
**F (p)**			8.063 (< 0.001*)	22.990 (< 0.001*)	38.844 (< 0.001*)
**Marital Status**					
Single	430	86.0	11.27 ± 5.32	23.16 ± 7.25	26.20 ± 9.61
Married	47	9.4	11.34 ± 5.13	22.83 ± 7.62	23.98 ± 11.31
Divorced	11	2.2	16.91 ± 5.19	25.55 ± 7.85	26.55 ± 12.34
Widowed	12	2.4	18.58 ± 3.12	26.67 ± 6.50	22.17 ± 12.58
**F (p)**			11.408 (< 0.001*)	**1.319 (0.268)**	**1.300 (0.274)**
**Type of Family**					
Nuclear	178	35.6	11.99 ± 5.46	23.52 ± 6.98	25.52 ± 9.91
Extended	322	64.4	11.34 ± 5.39	23.12 ± 7.46	26.11 ± 9.94
**t (p)**			**1.279 (0.201)**	**0.589 (0.556)**	**0.642 (0.521)**
**Father’s Educational Level**					
Illiterate	71	14.2	12.06 ± 4.23	25.24 ± 5.96	27.00 ± 8.43
Primary	99	19.8	12.29 ± 5.55	24.55 ± 6.38	28.48 ± 9.72
Secondary	184	36.8	11.59 ± 5.69	23.89 ± 5.95	27.59 ± 8.95
University or above	146	29.2	10.82 ± 5.45	20.65 ± 9.12	21.48 ± 10.55
**F (p)**			**1.713 (0.163)**	9.967 (< 0.001*)	15.163 (< 0.001*)
**Mother’s Educational level**					
Illiterate	124	24.8	11.51 ± 4.95	24.52 ± 6.30	27.56 ± 8.95
Primary	107	21.4	12.40 ± 5.49	24.29 ± 7.08	26.07 ± 10.22
Secondary	165	33.0	11.20 ± 5.39	22.60 ± 6.73	26.35 ± 9.39
University or above	104	20.8	11.38 ± 5.91	21.76 ± 8.97	23.03 ± 11.02
**F (p)**			**1.143 (0.331)**	3.942 (0.008*)	4.270 (0.005*)

Mpox: Moneybox; MFS: Monkeypox Fear Scale

*: Statistically significant at *p* ≤ 0.05 F = ANOVA test t = independent t-test

Table 2 reveals varied levels of concern and fear regarding Mpox among respondents. A significant majority, 56.2%, reported that they “never or rarely” considered their chances of being infected with Mpox. When asked how thinking about the possibility of infection affected their mood, again, 56.2% stated that it had little to no effect (“never or rarely”), 66.6% reported that they “never or rarely” considered Mpox affecting their ability to carry out day-to-day tasks. Regarding the general concern about infection, 46.4% expressed minimal concern (“never or rarely”). Worry about being infected was similarly distributed, with 48.8% indicating low levels of worry. Notably, 54.2% felt that worrying about Mpox was not their primary problem. Overall, the mean score for concern was 11.57 (5.42), suggesting that while some participants were concerned, a significant portion did not experience high levels of fear or worry regarding Mpox infection.

**Table 2:** Monkeypox concern among the participants (*n* = 500)

Items	Never or Rarely	Sometimes	Often	Almost All the Time
During the last week, how often have you considered your chances of being infected with Monkeypox?	281(56.2)	150(30.0)	48(9.6)	21(4.2)
During the past week, has thought about the possibility of being infected with Monkeypox affected your mood?	281(56.2)	137(27.4)	60(12.0)	22(4.4)
During the past week, have you considered the possibility of being infected with Monkeypox affecting your ability to carry out your “day-to-day” activities?	333(66.6)	101(20.2)	46(9.2)	20(4.0)
How concerned are you about being infected with Monkeypox?	232(46.4)	150(30.0)	78(15.6)	40(8.0)
How often do you worry about being infected with Monkeypox?	244(48.8)	159(31.8)	72(14.4)	25(5.0)
Is being worried about being infected with Monkeypox a major problem for you?	271(54.2)	134(26.8)	54(10.8)	41(8.2)
**Mean (SD)**	11.57	(5.42)		

Table 3 presents varied emotional responses to the disease. A noTable 40.4% of the respondents reported feeling neutral about their fear of Mpox. When considering discomfort associated with thinking about Mpox, 43.4% agreed that it makes them uncomfortable to contemplate the disease. Additionally, 45.4% of participants experienced physical symptoms, such as wet hands, when thinking about Mpox. Concerns about life-threatening implications were also present; 25.8% of participants strongly agreed that they were afraid of losing their lives to Mpox. The fear triggered by media coverage was evident, with 49.2% feeling nervous or anxious after seeing news about Mpox. Sleep disturbances due to worries about Mpox were reported by 19.2% of participants, indicating that a portion of the population experiences significant anxiety related to the disease. The mean score for Mpox fears was 23.26 (7.29), suggesting moderate participant fear.

**Table 3:** Monkeypox fears among the participants (*n* = 500)

Items	Strongly disagree	disagree	Neutral	Agree	Strongly agree
Am I terrified of Monkeypox?	18 (3.6)	64 (12.8)	202 (40.4)	91 (18.2)	125 (25.0)
Does it make me uncomfortable to think about Monkeypox?	22 (4.4)	95 (19.0)	166 (33.2)	109 (21.8)	108 (21.6)
Do my hands get wet when I think about Monkeypox?	21 (4.2)	70 (14.0)	182 (36.4)	107 (21.4)	120 (24.0)
Am I afraid of losing my life to Monkeypox?	26 (5.2)	104 (20.8)	141 (28.2)	100 (20.0)	129 (25.8)
When I see news and stories about Monkeypox on social media, do I get nervous or anxious?	19 (3.8)	64 (12.8)	171 (34.2)	117 (23.4)	129 (25.8)
I cannot sleep because I am worried about having Monkeypox.	49 (9.8)	180 (36.0)	115 (23.0)	60 (12.0)	96 (19.2)
Does my heart race when I think about getting Monkeypox?	47 (9.4)	161 (32.2)	122 (24.4)	64 (12.8)	106 (21.2)
**Mean (SD)**	23.26	(7.29)			

Table 4 presents respondents’ generally proactive approach to hygiene and safety measures. A significant portion, 38.6%, reported that they “often” wash their hands with soap and water. However, 21.2% indicated they “never” disinfect their hands with alcohol when soap is unavailable. Regarding avoiding contact with individuals exhibiting skin rashes, 31.6% of participants reported doing so “often,” and 18.6% said they “always” avoid such contact. Similarly, 29.8% of respondents indicated they “often” refrain from sharing clothes and other items when sharing personal items. Ensuring each family member has their cutlery was less common, with only 24.2% stating they do this “often.” Participants also demonstrated varying levels of caution when going out; 30.8% said they “sometimes” avoid touching their eyes, nose, and mouth. Notably, when dining out, a majority (34.4%) reported that they “never” clean cutlery with alcohol or lemon before use. 39.4% of participants stated they “never” wear masks when going outside, highlighting a significant area for improvement in preventive behavior during the ongoing concerns about Mpox transmission. Additionally, 30.6% indicated they “often” disinfect the bathroom after visitors use it. The mean score for preventive practices was recorded at 25.90 (9.92), suggesting moderate engagement in preventive behaviors among participants.

**Table 4:** Monkeypox Preventive practices used by the participants (*N* = 500)

Items	Never	Rarely	Sometimes	Often	Always
I wash my hands with soap and water	93(18.6)	27(5.4)	77(15.4)	193(38.6)	110(22.0)
I disinfect my hands with alcohol if I do not have soap handy	106(21.2)	79(15.8)	143(28.6)	126(25.2)	46(9.2)
I avoid contact with people who have skin rashes	102(20.4)	58(11.6)	89(17.8)	158(31.6)	93(18.6)
I avoid sharing clothes and other personal items	99(19.8)	55(11.0)	93(18.6)	149(29.8)	104(20.8)
At home, each person in the family has their cutlery	132(26.4)	79(15.8)	117(23.4)	121(24.2)	51(10.2)
When I go out, I avoid touching my eyes, nose and mouth	127(25.4)	70(14.0)	154(30.8)	107(21.4)	42(8.4)
When I go to a restaurant, I usually clean the cutlery with alcohol or lemon	172(34.4)	96(19.2)	120(24.0)	81(16.2)	31(6.2)
When I go outside, I use the mask	197(39.4)	105(21.0)	105(21.0)	69(13.8)	24(4.8)
I disinfect the bathroom of the house when a visitor uses it	101(20.2)	64(12.8)	91(18.2)	153(30.6)	91(18.2)
**Mean (SD)**	25.90	(9.92)			

Table 5 presents the correlation coefficients among participants between Mpox concern and preventive practices. The results indicated a strong statistically significant positive correlation between Mpox concern and fear (*r* = 0.646, *p* < 0.001). A moderate positive correlation existed between Mpox concern and preventive practices (*r* = 0.229, *p* = 0.001). The correlation between fear and preventive practices was also significant (*r* = 0.432, *p* < 0.001), suggesting that higher levels of fear are linked to more frequent engagement in preventive measures.

**Table 5:** Correlation coefficient between Mpox concern, fear, and preventive practices

Variables		Mpox concern	MFS	Mpox preventive practices
Mpox concern	r			
	p			
MFS	r	0.646*		
	p	< 0.001*		
Mpox preventive practices	r	0.229*	0.432*	
	p	0.001**	0.001*	

Mpox: Moneybox; MFS: Monkeypox Fear Scale

r: Pearson coefficient **. Correlation is significant at the 0.01 level (2-tailed)

Table 6 presents a multiple linear regression analysis demonstrating a significant overall fit, with an R^2 value of 0.216, indicating that the independent variables included in the model can explain approximately 21.6% of the variance in preventive practices. The F-statistic of 22.633 (*p* < 0.001) further confirms the model’s significance. Among the predictors, MFS emerged as a strong positive predictor of preventive practices. This relationship suggests that fear may motivate individuals to adopt more preventive measures against Mpox. Monkeypox concern also showed a positive but non-significant association with preventive practices (B = 0.138, *p* = 0.156), indicating that while concern may influence behavior, it does not do so as strongly as fear does in this model. Age did not significantly predict preventive practices (B = 0.251, *p* = 0.637), suggesting that age may not be crucial in influencing health behaviors related to Mpox. Family monthly income was significantly negatively associated with preventive practices (B = -1.885, *p* = 0.010), indicating that lower income levels are associated with less engagement in preventive behaviors. The educational levels of both parents did not yield significant associations with preventive practices, suggesting that these factors may not directly influence individual behaviors regarding Mpox prevention.

**Table 6:** Multiple linear regression model analysis for the association between Monkeypox concern, fear, and preventive practices

Variable	Unstandardized Coefficients (B)	Standardized Coefficients (Beta)	t	*p*	95% CI	
					LL	UL
Mpox concern	0.138	0.097	1.421	0.156	0.053	0.328
MFS	0.584	0.075	7.800*	< 0.001*	0.437	0.731
Age	0.251	0.530	0.473	0.637	-0.791	1.293
Family monthly income	-1.885	0.725	-2.600*	0.010*	-3.309	-0.461
Father’s Educational level	-0.819	0.548	-1.494	0.136	-1.897	0.258
Mother’s Educational level	0.186	0.485	0.383	0.702	-0.767	1.139
**R**^**2**^ = **0.216, Adjusted R**^**2**^ = **0.206, F** = 22.633^*^, ***p*** < 0.001^*^						

Mpox: Moneybox; MFS: Monkeypox Fear Scale

F: ANOVA test R^2^: Coefficient of determination t: t-test of significance

CI: Confidence interval LL: Lower limit, UL: Upper Limit *: Statistically significant at *p* ≤ 0.05

## Discussion

Our findings reflect a complex emotional landscape where fear exists but is not universally pervasive; many Egyptian nursing students experience varying degrees of concern and anxiety regarding Mpox, influenced by personal perceptions and external information sources. While many students were taking steps to protect themselves from Mpox, there were notable gaps in certain practices that could be addressed through targeted health education and interventions to enhance awareness and compliance with recommended preventive measures.

Our findings revealed that while some students had concerns, a notable number did not show elevated fear or anxiety regarding Mpox infection. These findings are consistent with other studies that have explored public perceptions of Mpox, highlighting a nuanced relationship between awareness, concern, and emotional reactions to the illness. For example, research conducted among healthcare professionals and medical students in Egypt indicated that 55.3% of the participants possessed sufficient knowledge about Mpox. In comparison, 44.5% and 39.8% demonstrated positive attitudes and perceptions towards the disease. Analysis using binary logistic regression showed that a favorable attitude was notably associated with male participants (*p* = 0.045), those living in urban areas (*p* = 0.002), and nurses (*p* = 0.002). This indicates that despite some knowledge, many individuals may not perceive Mpox as a severe threat that would induce significant fear or anxiety [30]. Similarly, research from the United Arab Emirates reported that while 54% of participants were worried about Mpox during an outbreak, a significant number still maintained ambivalence towards the disease. This reflects a broader trend where knowledge does not always equate to heightened fear; individuals may rationalize their concerns based on their understanding of the disease’s transmission and severity [31].

Our results found a strong positive correlation between Mpox concern and fear, suggesting that higher levels of concern about Mpox are associated with increased fear of the disease. Additionally, a moderate positive correlation existed between Mpox concern, fear, and preventive practices, indicating that individuals expressing more significant concern and fear about Mpox are likelier to engage in preventive behaviors. This relationship may reflect a behavioral response where fear motivates individuals to take precautionary actions to protect themselves from potential infection. This relationship underscores the psychological impact that concern can have on individuals, potentially influencing their emotional responses to the threat of infection. Research supports this correlation, indicating that fear often acts as a motivator for health-related behaviors. For instance, a study conducted in Peru found that greater emotional fear of Mpox was positively related to the intention to vaccinate against the disease [32]. This aligns with the Theory of Planned Behavior (TPB), which posits that psychological constructs like fear can influence individuals’ intentions to engage in preventive health behavior [33]. Similarly, during previous infectious disease outbreaks, heightened fear levels among undergraduate nursing students and intern nurses have been linked to increased compliance with health recommendations, such as vaccination and adherence to preventive measures [34, 35].

Moreover, the emotional impact of concern and fear is compounded by media exposure and personal experiences with past pandemics. For example, individuals who have previously experienced anxiety during outbreaks may be more susceptible to heightened fears about new diseases like Monkeypox. Studies have shown that anxiety symptoms can exacerbate emotional reactions to health threats, leading to a cycle of increased worry and fear [34, 36]. In this context, the media’s portrayal of Mpox can amplify fears, particularly when coverage emphasizes potential risks without providing balanced information on prevention and management strategies.

The findings from our multiple regression analysis emphasize the critical role of fear as a primary motivator for engaging in preventive practices against Mpox. While concern about Mpox also demonstrated a positive but statistically non-significant association with preventive behaviors, it is evident that fear exerts a more substantial influence in this context. This aligns with the Protection Motivation Theory (PMT) proposed by Rogers [37], which posits that fear is vital in shaping health-related behaviors. According to PMT, individuals are motivated to protect themselves when they perceive a threat, and this perception is mediated by cognitive evaluations of the risk and their efficacy in responding to it. Research in health psychology supports the notion that emotional and cognitive factors drive health behaviors. For instance, Ferrer and Klein [38] they have highlighted that emotional variables, including fear and anxiety, significantly influence individuals’ actions regarding health risks. Hayes, Luoma [39] proposed that emotional factors are more effective in motivating behavior change.

The analysis of socioeconomic factors found that family monthly income was significantly negatively associated with preventive practice. This finding aligns with Ahmed, Abdulqadir [23], who emphasized that individuals from lower socioeconomic backgrounds often face significant barriers to accessing health information and services, which can hinder their ability to engage in preventive practices. By addressing these socioeconomic factors, public health initiatives can better equip communities to respond effectively to emerging health threats, thereby reducing the overall impact of diseases such as Mpox while addressing the significant psychological impact among nursing students [40–42].

### Strengths and limitations

This study has several strengths, including its focus on a timely and essential public health issue, nursing students’ concerns, and preventive practices regarding Mpox. By assessing concerns and preventive behaviors, the study provides valuable insights into future healthcare professionals’ preparedness and awareness levels, contributing to the existing knowledge in outbreak response education. The relatively large sample size enhances the reliability of the findings, while the use of validated questionnaires ensures the consistency and accuracy of the collected data. However, there are limitations to consider. The study’s cross-sectional nature prevents the establishment of causal relationships, limiting the ability to infer how students’ perceptions may evolve. Additionally, the reliance on self-reported data may introduce bias, as participants could respond in a socially desirable manner rather than accurately reflecting their true beliefs or behaviors. Finally, the study’s findings may not be generalizable beyond the specific population sampled, which could limit the applicability of the results to other nursing student populations or healthcare settings.

## Conclusion

The current survey underscores the importance of understanding how fear and concern regarding Mpox influence preventive practices among undergraduate nursing students. While fear can act as a strong motivator for engaging in protective behaviors or adherence to health guidelines, concern may not always translate into action unless an adequate level of fear accompanies it. The findings indicated that concern and fear regarding Mpox vary significantly across demographic factors, with younger individuals generally exhibiting lower fear levels than older age groups. Preventive practices tend to increase with age, and individuals with higher family monthly incomes engage more in health-protective behaviors.

### Nursing implications

The findings suggest important implications for nursing education, research, and practice. In nursing education, the results indicate a need to emphasize the role of socioeconomic factors and fear in shaping health behaviors. Curriculum enhancements could include training nursing students to recognize how financial constraints and emotional responses influence preventive health practices and preparing future nurses to provide more tailored education to diverse populations. Additionally, the observed correlation between fear and preventive practices underscores the importance of integrating psychological considerations into infection prevention education, helping nurses develop strategies to address patients’ fears in a way that promotes proactive health behaviors.

For nursing research and practice, the data reveal opportunities to explore targeted interventions to enhance preventive behaviors, especially among individuals with lower income levels or higher levels of concern. The significant correlation between fear and preventive measures suggests that fear-based educational campaigns may effectively increase adherence to protective practices. However, these should be carefully designed to avoid causing undue anxiety. Furthermore, practice-based initiatives could focus on increasing accessibility to preventive resources for economically disadvantaged populations, such as providing hand sanitizers or masks. Addressing these disparities could lead to more equitable health outcomes and better preparedness for future outbreaks.

## Data Availability

The datasets generated and analyzed during the current study are not publicly available due to confidentiality agreements but are available upon reasonable request from the corresponding author.
